# Medication dosage calculation among nursing students: does digital technology make a difference? A literature review

**DOI:** 10.1186/s12912-022-00904-3

**Published:** 2022-05-23

**Authors:** Kerstin Stake-Nilsson, Malin Almstedt, Göran Fransson, Davoud Masoumi, Annika Elm, Monique Toratti-Lindgren, Annica Björkman

**Affiliations:** 1 Department of Caring Science, Faculty of Health and Occupational Studies, University of Gävle, 801 76 Gävle, Sweden; 2 The Library, University of Gävle, Gävle, Sweden; 3 Department of Educational Science, Faculty of Business and Economics Studies, University of Gävle, Gävle, Sweden

**Keywords:** Medication dosage calculation, Digital technology

## Abstract

**Background:**

Patient safety is a major part of nursing care and following patients’ medication orders is considered one of the greatest responsibilities of individual nurses and nursing Failure to make safe drug calculations poses serious risks to patient safety. It is therefore important to strengthen nursing students’ numeracy skills and conceptual abilities during their education. Research suggests that digital technologies play an increasingly important role in promoting nursing students’ knowledge and medication dosage calculation (MDC) skills. The present review aims to identify and critically evaluate research investigating how the use of digital technologies informs the development of nursing students’ MDC skills.

**Methods:**

A systematic literature review was performed within Scopus (Elsevier), Academic Search Elite (Ebsco), Cinahl (Ebsco), ERIC (Ebsco), Web of Science and PubMed. Research papers on MDC using digital technologies were considered for inclusion. Starting from 2843 sources, eighteen research articles met the inclusion criteria.

**Results:**

The results show that use of digital technologies can reduce nursing students’ medication errors. Interestingly, web-based courses were the most commonly used digital technologies aimed at developing nursing students’ MDC skills. However, such courses had limited impacts the development of these skills.

**Conclusion:**

The present review concludes by mapping the current knowledge gaps and making suggestions for further research.

**Supplementary Information:**

The online version contains supplementary material available at 10.1186/s12912-022-00904-3.

## Background

Patient safety is a major component of nursing and following patients’ medication orders is one of the most important things nurses do [1–3]. Several studies [4–6] emphasized the difficulties nursing students face when calculating medication doses. More than half of nursing students around the world fail numeracy and medication dose calculation (MDC) assessments, indicating the global nature of this issue [6].

MDC mistakes are also one of the most common complications in modern medicine [7]. Many of the errors are avoidable and constitute a significant risk to patient safety [8]. These errors can have a variety of consequences for patients, ranging from loss of a medication’s beneficial effects to death. Aside from the ethical issues, MDC errors can result in higher healthcare expenses [9]. Medical errors are expected to cost at least 20 billion dollars in the United States [10] and in Sweden around SEK 8 billion per year [11].

According to Karlstedt, Wadensten, Fagerberg, and Pöder [12], nursing education does not provide sufficient competence in this field. Newly registered nurses are expected to be proficient in implementing safe pharmacotherapy immediately after graduation. According to nursing studies, nurses have inadequate MDC skills, and this is a global concern for the nursing profession [13, 14]. Evidence also suggests that nursing students lack a solid numeracy foundation [15–17] and have had problems with mathematical competency for more than 40 years [15, 16]. Several studies conducted since the 1980s have discovered that a large proportion of nursing students are unable to pass arithmetic assessments [16, 18, 19]. As a result, it is critical to design learning strategies for recognizing the hurdles to success as well as to provide chances for nursing students to improve their numeracy skills.

MDC has always been taught with a chalkboard, pencil, and paper. More recently, a slew of studies investigating the use of novel approaches to teaching medicine administration in simulation have revealed that such methods are already ubiquitous. Several investigations [20, 21] have found that employing technology such as calculators, web-based education, simulations, forums, and Virtual Learning Environments improves nurses’ knowledge and abilities in nursing operations. Despite these advancements in technology, nursing students continue to have difficulty with numerical computation. To overcome these issues, various teaching methodologies have been explored, but no single system has been shown to be extremely effective.

The goal of this study is to find and critically assess the literature on how the usage of digital technologies influences the development of MDC skills in nursing students. Better MDC teaching recommendations for nursing students can be developed by gaining insights into the types of technologies that are being employed and their consequences.

## Method

The search process was initiated early by discussing different concepts within the research group, thereafter, delimiting relevant search terms. One of the authors (MAJ), a research librarian, undertook the literature search, but then the whole research team considered the evidence against exclusion and inclusion criteria regarding the overall topic in relation to nursing.

An early scoping search indicated that the phrase “information and communications technology” (ICT) was not sufficient to retain studies relevant to the present aim. ICT is a generic term for a multitude of applications concerning digital technology; it is used in a variety of fields and has different meanings. Therefore, other relevant terms representing different aspects of ICT were identified and included in the search strategy. The terms included in the search strategy are presented in Table 1. The terms were combined using Boolean operators and based on the principle (population OR population) AND (exposure OR exposure) AND (outcome OR outcome).

**Table 1:** Terms (free text) included in the search

Population	Exposure	Outcome
“nurs* train*”	“comp* based learn*”	“drug calcula*”
“nurs* program*”	“comput* assist* instruct*”	“dos* of drug*”
“nurs* student”	“comput* assist* learn*”	“drug administ*”
“nurs* educat* research”	“comput* learn*”	“mathem* skill*”
“student nurs*”	“digit* game”	“medic* compet*”
“nurs* pupil”	“distance educat*”	“medic* error*”
“nurs* diploma program*”	“educat* game”	“medic* safe*”
“nurs* educat*”	“educat* strateg*”	“numer* skill*”
“pupil nurs*”	“educat* techn*”	“drug prepar*”
	“electron* learn*”	“administ* of medic*”
	“game-based”	“calcula* skills”
	“information* and commun* technolog*”	“dos* calcula*”
	“information* technol*”	“medic* calcula*”
	“interact* learn*”	“medic* document*”
	“learn* labora*”	
	“medic* game”	
	“non-tradit* educat*”	
	“nurs* intervent*”	
	“nursing inform*”	
	“open learn*”	
	“role-play”	
	“self directed learn*”	
	“technol* enhanced”	
	“web based”	
	“virtual learn*”	
	“virtual reality”	
	“personal digital assistant”	
	calculation	
	calculator	
	computer*	
	digital*	
	e-learn*	
	game	
	handheld	
	ICT	
	interactive	
	mobile	
	multimedia	
	online	
	simulat*	
	smartphone	

**Fig. 1:**
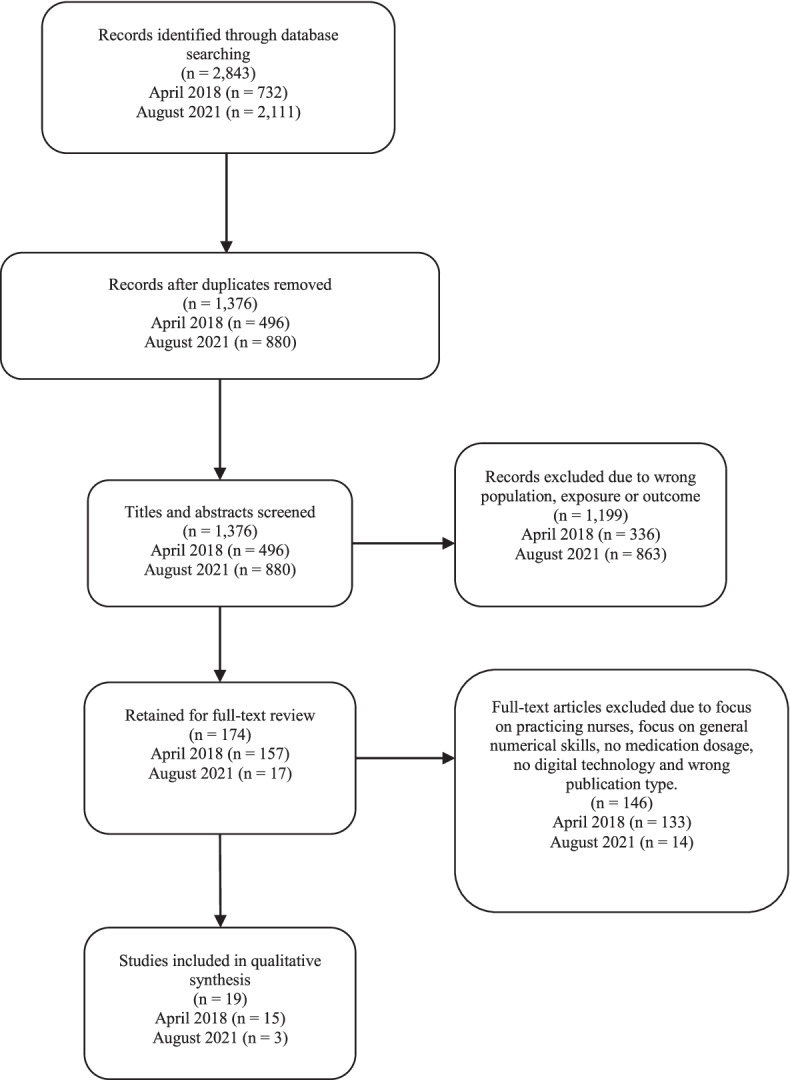
Search and inclusion process

The following databases were utilized: Scopus (Elsevier), Academic Search Elite/Premiere (Ebsco), Cinahl (Ebsco), ERIC (Ebsco) and Web of Science (Thomson Reuter). MAJ performed the searches in April 2018 and again in August 2021. In the second search the database PubMed was included to broaden the search base. The complete search and inclusion process are described in a PRISMA flow chart (Fig. 1), and the search strategies are available as supplementary material. The search strategy was initially created in Scopus, using free text and a step-by-step method. The strategy was subsequently adapted to the other databases. The search was limited to peer-reviewed articles, both original and review articles. Only articles written in English were included. No citation searches were performed.

The formal searches retained 2843 articles (Scopus *n* = 831, Academic search elite/premiere *n* = 321, Cinahl *n* = 796, Web of Science *n* = 218, Eric *n* = 24, and PubMed = 653). Duplicates were removed using Endnote and the de-duplication method suggested by Bramer, Giustini, de Jonge, Holland and Bekhuis [22]. After de-duplication, 1376 articles remained. For the number of articles retrieved and screened for each search occasion (2018 and 2021) see the PRISMA flow chart in Fig. 1.

Prior to the screening process, a test screening was performed, in which all authors screened the title and abstract of 30 articles to ensure consistency in the process. The test screen showed discrepancies among the research group, which led to further clarification of the inclusion and exclusion criteria.

The screening process was performed in three steps. In the first step, the authors – using the web-based tool Rayyan (rayyan.qcri.org) – screened all titles and abstracts using the inclusion and exclusion criteria (Table 2). The screening was performed in teams of two, consisting of one researcher from educational science and one from medical and nursing science, thus ensuring that both perspectives were included. Each team member independently screened all titles and abstracts assigned to them. If the results showed any conflicts, i.e., uncertainties regarding inclusion, the team members discussed the papers in question before a final decision was reached. In the first step, 1199 articles were excluded, leaving 174 eligible for the second step.

**Table 2:** Inclusion and exclusion criteria

	Inclusion	Exclusion
**Population**	nursing students, nursing education (or variants of the term/ phrase)	studies focusing solely on any kind of practicing nurses any other type of health or medical students involved in drug/medication calculation, e.g., pharmacy students
**Exposure**	ICT or some kind of (digital) technology as a means of learning (regardless of storage medium) incl. Calculators	studies focusing solely on general pedagogical/didactic aspects that are not linked to ICT
**Outcome**	drug calculation, medication calculation (or variants of the term/phrase)	studies focusing solely on general improvement of numerical or mathematical skills

In the second step, the full texts of the eligible articles were reviewed. The 174 articles were divided equally among the team members, ensuring that they did not read the articles they had screened in the first step. Each team member independently read the full articles. Any uncertainty concerning including or excluding an article was first discussed by the team members and later by all authors. The last discussion showed some minor differences, leading to a second assessment of a selected number of articles. Of the 174 articles, 146 were excluded for focusing on the wrong population, exposure or outcome, leaving 19 eligible for qualitative synthesis.

In the third step, each author separately read eight articles, grouping the main findings in a joint matrix. To ensure uniform presentation, one author (KSN) performed the compilation of all separate matrixes.

## Results

Eighteen research articles met the inclusion criteria in that they addressed the issue of developing nursing students’ MDC skills using digital technologies. An outline of the designs, approaches, aims, and samples of the included articles are presented in Table 3. The findings entail a narrative description of the results of the included papers and are presented as follows: Descriptions of the various digital tools used in MDC and their effects. The digital tools are: calculators (*n* = 3), online tutorial (*n* = 1), personal digital assistants (*n* = 4), web-based courses (*n* = 8) and simulations (*n* = 2). Most studies had a descriptive or experimental design.

**Table 3:** Summary of included articles; digital technologies used in medication dosage calculation (MDC)

Author(s) and year	Study design, aim and samples
Aydin and Dinç (2017) [20]	Comparative design. Quantitative approach. To evaluate the effectiveness of web-based instruction in improving arithmetical and MDC skills, N 63.
Cunningham and Roche (2001) [23]	Descriptive design. Quantitative approach. To determine competence in MDC through an online test, N 52.
Craig et al. (2021) [24]	Experimental design. Quantitative approach. To determine whether errors could be reduced using ‘real-life’ situations, simulation. N 80.
Edwards et al. (2019) [25]	Descriptive design. Collaborative approach. To determine whether errors could be reduced using ‘real-life’ situations, simulation. N 16.
Glaister (2005) [26]	Comparative design. Quantitative approach. To determine the effect of different instructional approaches to knowledge acquisition regarding dosage calculation, N 9.
Greenfield (2007) [27]	Experimental design. Quantitative approach. To determine whether errors could be reduced using personal digital assistant technology, N 87.
Grugnetti et al. (2017) [28]	Comparative design. Quantitative approach. To verify whether calculator use reduces errors, N 78.
Hitam (2020) [29]	Experimental design. Quantitative approach. To compare web-based instruction and traditional classroom learning in decreasing the number and types of errors in MDC. N 120.
Maag (2004) [30]	Experimental design. Quantitative approach. To determine the effectiveness of an online interactive multimedia learning tool, N 96.
Mackie and Bruce (2016) [4]	Descriptive design Quantitative approach. To determine whether online interventions are effective in decreasing the number and types of errors in MDC, N 65.
McMullan et al. (2011) [31]	Comparative design. Quantitative approach. To compare an interactive e-drug calculations package with traditional handout learning support, N 229.
Pereira et al. (2016) [32]	Experimental design. Quantitative approach. To evaluate the influence of the use of digital applications in learning about MDC, N 100.
Ramjan et al. (2014) [33]	Mixed methods design. Quantitative approach. To identify strategies that help nurse academics tailor their drug calculation teaching, N 390.
Shockley et al. (1989) [34]	Experimental design. Quantitative approach. To determine the effect of using calculators on an MDC examination; N 166.
Tarnow and Werst (2000) [35]	Descriptive design. Quantitative approach. To examine the effectiveness of a calculator regarding scores on a drug calculation examination, N 85.
Valizadeh et al. (2016) [36]	Experimental design. Quantitative approach. To measure individuals’ knowledge of drug prescription principles through an e-learning program, N 82.
Van et al. (2016) [37]	Experimental design. Quantitative approach. To evaluate an e-learning course compared to face-to-face lectures, N 411.
Wright (2008) [38]	Experimental design. Quantitative approach. To test the effectiveness of a range of strategies in improving retention of drug calculation skills; N 172.

### Calculators

Three studies looked at arithmetic and conceptual skills in MDC examinations [28, 34, 35].

Grugnetti et al. [28] randomly assigned second-year students to one of two groups: one with a calculator and the other without. The results revealed a marginally significant between-group difference (t = 1.78, *p* = 0.078, IC al 95% 0.18-3.33). Using a calculator did not aid nursing students in solving conceptual problems, but it did somewhat increase their performance.

Concerning bachelor’s level nursing students, Shockley et al. [34] utilized the same strategy as Grugnetti et al. [28]. The results showed that calculator use was linked to more conceptual errors but fewer arithmetic errors.

Tarnow and West [35] performed a second replication on students in their first semester and found no significant differences.

### Online tutorial

Wright [38] studied two groups of nursing students using an online lesson. An MDC skills workshop, medication calculation books, an online math tutorial, and a face-to-face math tutorial were given to the intervention group (I). Only lectures on drug calculating abilities were given to the control group (C). The results revealed that the procedures used in Group I resulted in a significant difference in retention of medication calculating skills (U = 39.5, *p* 0.001).

### Personal digital assistant

Glaister [26], Greenfield [27], McMullan, Jones, and Lea [31], and Pereira, Caetano, Frota, and da Silva [32] are four studies that have concentrated on the use of various personal digital assistants (PDA) technology devices.

In Glaister’s [26] study, second-year nursing students were assigned to one of three instructional modalities: computerized learning (CL), integrative learning (IL), or a combination of the two. CL provided an option for autonomous learning using computer software that provided rapid feedback. IL consisted of two one-hour tutorials in which pre-intervention guided study modules were used to strengthen procedural and declarative knowledge. Both IL and CL were given to the third group. Regarding transfer measures and knowledge acquisition, the results demonstrated no statistical difference between the three techniques, while the difference for procedural knowledge measures was significant (F (2, 47) = 3.33 at p.044). CL was highly effective in increasing procedural knowledge, according to a post hoc test (alpha = 0. 10).

Greenfield [27] examined junior and senior bachelor’s level nursing students. Clinical judgments based on medicine administration and calculations were part of the test. Students were self-selected to either a control group (C), equipped with textbooks and reference books found in most clinical units, or the experimental (PDA) group, equipped with a drug program created for healthcare providers. The variables accuracy and speed were tested. Each erroneous answer received a score of 0 and each good answer received a score of 1. Group C had a mean accuracy score of 3.5, while Group PDA had a score of 4.1 (*p* = 0.037). Each participant’s elapsed time was recorded using a stopwatch. Group C (mean = 17.2 minutes) worked slower than Group PDA (mean = 13.2 minutes) (*p* = 0.002). The findings showed that PDAs have the potential to improve patient safety by boosting the accuracy and speed of healthcare delivery.

McMullan et al.’s [31] study compared traditional ‘handout’ learning support (Group C) with an interactive e-drug calculations package, which included drug calculation skills, self-efficacy, and satisfaction with the support material (Group I). Before and after a 12-week clinical practice placement, students took part in the study halfway through their second year. The ability to calculate drugs improved significantly in Group I (September: mean 41.2% (SD = 18.9) versus 48.4% (SD = 18.1); t = 2.92; *p* = 0.007. In February, the average was 36% (18.3) compared to 47.6% (SD = 13); t = 3.34; *p* = 0.003). Students in Group I were considerably better at making drug calculations after the intervention than students in Group C (September: mean 48.4% (SD = 18.1) versus 34.7% (SD = 20.8); t = 2.29; *p* = 0.027. In February, the mean was 47.6% (SD = 13.0) versus 38.3% (SD = 14.7); t = 2.34; *p* = 0.024).

Pereira et al. [32] divided second-semester students into two groups: a control group (C) that used a calculator and prior math skills, and an intervention group (I) that used the CalcMed app. Both groups were evaluated before and after the teaching technique was implemented, with questions concerning medication calculations included in the testing. Regarding measurement errors, accurate answers, and test time resolution, Group C scored 5.02 compared to 8.14 in Group I. Group C’s average test execution time was longer than Group I’s (38.9 versus 15.7 minutes). Individuals who used the program to complete the calculations had considerably superior usage of the variable error average, average test run, and mode hits, with a percentage success rate in the region of 80% for the recommended items. Group C had a mean score of 5.02 3.21 points, whereas Group I received an average score of 8.14 1.67 points, indicating that the program was used more efficiently.

### Web-based courses

Valizadeh et al. [36] and Van et al. [37] compared the efficacy of face-to-face and e-learning courses. Valizadeh et al. [36] used a questionnaire to examine students in their second and third semesters on their understanding of drug administration principles and capacity to perform medicinal calculations. Students were divided into two groups: control (lecturing) and education with study-specific software. After 4 weeks of training, the ability and knowledge posttests were conducted using the same items. There was no significant difference between the two groups in medicinal calculation ability (*p* > 0.05), but medicine calculation skills rose significantly in both groups following training (*p*0.05). Both training approaches had no significant influence on study participants’ knowledge of medicinal principles (*p* > 0.05), whereas the control group’s score on knowledge of medicinal principles grew non-significantly.

Van et al. [37] looked at nursing students in their last year. The experimental group (I) received face-to-face lessons, whereas the control group (C) received an e-learning course that included an introduction to medicine calculation, dosage calculation, general exercises, and case-based exercises. Prior to the course, shortly after the course, and 3 months later, both groups conducted a validated medication calculation exam. The results showed that students in both groups improved considerably in medication calculation when tested immediately and 3 months later (Group C: F (2.448) 109.98, P 0.001, Group I: F (2.431) 41.61, P 0.001). Between the exams given immediately after the course and 3 months later, the drop seen in Group C was not significant (median 0.13; 95% CI 0.21–0.46; *p* = 0.458). After 3 months, the results for Group C leveled out, with a substantial drop from immediately after the course to 3 months (median 0.62; 95% CI 0.27-0.97; *p* = 0.001).

The effectiveness of web-based intensive training has been tested in research by Aydin and Dinç [20] and Mackie and Bruce [4]. Second-, third-, and fourth-year students increased their arithmetical and MDC skills as measured by the Demographic Information Form (DIF) at pre-and post-tests, according to Aydin and Dinç [20]. Students received 8 weeks of web-based training, which included quizzes, audio presentations of lectures, and online posttests. The students were not allowed to use calculators. The results showed that only five students (7.9%) had scores above 90 on the arithmetic skills pretest, but this number climbed to 19.1% on the posttest. No student scored higher than 90 on the MDC skills pretest, while 41.2% did so on the posttest. The pretest mean score for arithmetic skills was 74.98 ± 12.14; the posttest mean score was 82.03 ± 9 (*p* = .000). Similarly, the MDC skills pretest mean score was 71.55 ± 12.29 which grew 14.42 points on the posttest, reaching 82.03 ± 9 (*p* = .000).

Mackie and Bruce [4] looked at whether online intervention options for nursing students were successful in reducing the number and types of MDC errors. The results of the summer term’s pretests were then compared to the following year’s posttests. Nursing students responded positively to an intervention that included practice opportunities, online conceptual learning opportunities, and simulations demonstrating the effects of MDCs, as measured by pre- and posttests. On the MDC tests, conceptual and procedural errors were more common than unit problems (8.3% vs. 6.1% vs. 1.8%). On the pretest, the most prevalent conceptual error was “No Answer,” which indicated that no effort had been made to provide an answer. Multiplication and division were the most prevalent procedural errors. There were additional lessons on reviewing fractions and decimals, as well as lessons on converting between SI units. The posttest test data revealed that all three categories of errors (conceptual, procedural, and unit related) were still occurring. Compared to students who took the pretest, students who took the drug calculation posttest made fewer errors in all the error categories.

Cunningham [23] supported students’ learning through a WebCT platform. For senior nursing students, a program was implemented that included medication calculation education as well as activities such as studying a medication dose book, taking an online official test, and attending a math review session. Students could also use the Web-CT platform to take a practice MDC test and participate in one-on-one teaching sessions. There were no calculators allowed. Students could take the practice test at any time and from any location, and they could take it as many times as they wanted. Students could take a secure quiz using an Internet-accessible platform. Students could practice computerized testing for the licensing exam and receive feedback on their responses to help them learn better. By the second retake test, every student had a score of at least 90%. This group had taken the regular curriculum the previous semester, and it took five retakes for the full set of students to pass with a 90% pass rate. There was a statistically significant difference in performance outcomes across the groups (P 0.05).

Online learning help was employed by Maag [30] and Ramjan et al. [33]. Maag’s [30] study compared the effectiveness of an online interactive multimedia learning tool to text alone in boosting math self-efficacy, math achievement, and student satisfaction among undergraduate nursing students. The four treatment groups each lasted 1 h. Group 1 read three text-based mathematical modules and learned from them. Group 2 read the identical modules as Group 1, but with the addition of visuals. Group 3 saw the same modules as Group 1 and 2, but they also saw three multimedia modules. In addition, Group 4 saw three multimedia modules that included images, text, animation, and interactivity. Students in the interactive multimedia group displayed equal posttest and retest math knowledge, as well as the same math self-efficacy score as students in the control groups. Students in the interactive multimedia group were happier with their learning technique, using terms like “interesting” and “enjoyable” to describe it. The difference, t = 0.31, *P* = .76, and t = 1.00, *P* = .32, respectively, were insignificant. Students’ learning was not hampered by computer-based learning modules, according to the findings.

Ramjan et al. [33] compared the performance and views of second-year nursing students on a diagnostic math paper with only questions vs. a diagnostic math paper including visual images and questions. Simulated medicine calculation scenarios, online practice quizzes, paper-and-pen examinations, visually enhanced didactic remediation, and hands-on contextualized workshops were among the interventions; scores improved significantly from Test 1 (decontextualized) to Test 2 (contextualized). The contextualized paper was selected by most students (80%). Students liked the visual images as well, indicating that they lowered tension and anxiety and allowed for “deeper learning” of numeracy abilities. Five statistically significant factors were included in the analysis by the researchers: enrollment status (international), previous mathematics education (lower than Year 10 math), online practice quiz attempts (4 counts or more), overall online practice quiz grades (59% or more) and felt confidence (ratings of 8 out of 10 or more). Being an overseas student (OR: 2.91; 95% CI: 1.31, 6.45) was the best predictor of good performance on the initial numeracy skills exam, followed by the overall online practice quiz grade (59% or higher) obtained prior to the initial numeracy test (Test 1; OR: 2.55; 95% CI: 1.49, 4.38). The perception of confidence among students came in second (OR: 2.56; 95% CI: 1.49, 4.41).

Hitam [29] investigated the utilization of web-based instruction (WBI) among 122 second-year students. Based on the results of a mathematics diagnostic test, the students were divided into two groups: Control (C) and Experimental (E). After the theory on Drug Dose Calculation (DDC) was finished, a pretest using a modified intravenous additive test was performed. Group E received DDC utilizing WBI tutorials, whereas Group C received conventional classroom learning. After the intervention phase, Post-test I was conducted, and Post-test II was conducted 11 months later. The results showed a significant improvement in DDC skills between the groups, with Group E outperforming Group C, *p* = 0.01. There was no significant difference in the rate of DDC skill retention between the two groups.

### Simulation

The usefulness of employing simulation to engage students early in safe medication management practice has been evaluated by Edwards et al. [25] and Craig et al. [24].

Edwards et al. [25] built up four separate patient scenarios for second- and third-year students’ medication management simulations. The simulations were created to be student-led at the bedside, with fellow students playing patient roles with guides and some cue questions to help them confront the student nurses and make it more realistic. Debriefing took place at the conclusion of each medication management simulation. Students oversaw the debriefing by identifying their own strengths and limitations, as well as analyzing and synthesizing their emotions and behaviors. As a result of the students’ evaluations, four themes emerged: learning in the past, present and future, feeling challenged, sensing the experience in the moment, and leading in the moment. The medication management simulation proved effective in training future practitioners to advocate for safer medication management practices while also advancing the recognition of nurses’ autonomy.

Craig et al. [24] investigated the impact of a simulation program with integrated technology on medicine administration knowledge, competency, and confidence. Eighty-three third-year students were randomly assigned to one of two groups: intervention (I) or control (C). Group C received normal training, whereas Group I received additional clinical simulation experience as well as debriefing sessions on pharmaceutical safety practices. The Medication Safety Knowledge Assessment (MSKA) was used to assess participant knowledge, and the Medication Safety Critical Element Checklist was used to assess competency (MSCEC). Both groups’ post-test knowledge ratings improved, but the results were not statistically significant (*p* = 0. 05). Students who received the drug safety improvement intervention fared considerably better in a subsequent simulation than did students who had never participated in a simulation before (*p* < .001).

## Discussion

The results showed that web-based courses (*n* = 8) were the digital technologies most frequently used to develop nursing students’ MDC skills. However, these web-based courses had limited impacts. A number of the studies reported no differences between the intervention group and the control group. For instance, Van et al. [37] and Valizadeh et al. [36] reported no significant difference between the two groups in terms of medicinal calculation ability after training. Similar findings were reported by Mackie and Bruce [4], who presented posttest data indicating that all three types of errors (conceptual, procedural, and unit errors) were still being made by students. Students in Maag’s [30] intervention group demonstrated equal posttest and retest knowledge of math, and their math self-efficacy scores were the same, hence the intervention did not impede students’ learning.

On the other hand, a number of studies highlighted positive effects on students’ medication calculations. Cunningham and Roche [23] showed how all 52 students achieved a score of 90% by the second retake test. The group had participated in the standard program the preceding semester, at which time it took five retakes for the entire group of students to pass with a score of 90%. The intervention results indicated that there was a statistically significant between-group difference in performance outcomes. Their intervention consisted of practice MDC tests, where students were using the Web-CT platform and one-on-one tutorial sessions. The program provided students with feedback on answers to enhance learning, and students could access it as often as they wished. Because it was web-based it was easy to access. Perhaps the positive findings can be explained by the feedback given to students, e.g., increasing students’ understanding of the problem, or by the fact that the students using the Web-CT platform had ‘participated in the standard program the preceding semester.’ Students learning can be shallow, when they only manage the demands and goals of the course, or they can be immersive, encouraging them to apply a deep-learning approach [39]. Within an immersive learning approach, students can gain an understanding and ability to relate their ideas to previous knowledge and recognize patterns. In summary, only a few studies using a web-based intervention to develop students’ learning of MDC skills showed significant differences between the intervention group and control group. Based on these findings, it is reasonable to wonder why the studies used different kinds of educational interventions, but only one [23] could show a positive effect. The findings of Ramjan et al.’s [33] study, similarly, highlighted the importance of providing visually enhanced ‘hands-on,’ ‘authentic’ and clinically contextualized interventions for numeracy learning.

Calculators have been used for decades to improve students’ MDC, and Hembree and Dessart’s [40] meta-analysis showed how use of calculators improves students’ basic skills regarding both problem-solving and accuracy. However, in nursing education, MDC has traditionally been taught using paper and pencil, despite developments in other curricula where calculators are taken for granted [41]. In our literature review, three studies [28, 34, 35] examined the effect of calculators on students’ mathematical skills. Only one of them [34] could show that calculator use was associated with fewer arithmetic errors, but with more conceptual errors. The others [28, 35] showed no difference between students using and those not using calculators. However, Grugnetti et al. [28] demonstrated how nursing students overall had significant gaps in mathematical competence. This is an interesting finding. Do nursing students as a group has gaps in their mathematical competence? Several studies have highlighted how educators are continually confronted with nursing students who lack basic mathematical skills [42–44]. The absence of effects of implementing different mathematical aids, such as calculators, could be explained by the fact that though calculators are used to facilitating students’ calculations, they require the user to have sufficient basic mathematical knowledge [41].

Four studies [26, 27, 31, 32] assessed whether PDA technology (such as hand-held computers, iPods, etc.) could reduce nursing students’ medication errors. Greenfield [27] showed that use of PDAs has the potential to reduce nursing students’ calculation errors. Similar findings were presented by Pereira et al. [32], who showed that introducing nursing students to the application CalcMed and using CalcMed led to significantly fewer errors among students. Previous studies in other teaching contexts [45] have shown that ICT can enhance students’ mathematical abilities [46]. Some of the studies evaluating the effect of different PDAs have addressed cell phone use in higher education [46], but many studies focused on lower levels of education, such as kindergarten [47] and compulsory school [48]. This focus may be based on technological developments and the recent trends in many Western countries where every pupil at school receives a personal portable computer or tablet PC [49] – a phenomenon that, in turn, has attracted the attention of researchers.

The method of simulation has been used more frequently in recent years [24, 25], and it seems that simulation is the method that has the best effect on students’ learning. This is an area that needs to be explored further using larger intervention groups and clearer randomizations. In addition to this, we need to examine students’ prior knowledge of mathematics. If we combine a mapping assessment of each student’s mathematical knowledge, based on which we can improve this knowledge, with the best learning tools, then perhaps we can near our ultimate goal: safe medication and, thereby, safe patient care.

Methodologically, twelve of the 18 analyzed studies are randomized experimental designs with interventions and control groups, and in one the students self-selected their group depending on whether or not they had a PDA [27]. Four studies [20, 23, 33, 34] did not use control groups but whole-group approaches and one was a pilot study aimed at designing an intervention study [4], while Wright [38] used two groups in a quasi-experimental approach, never mentioning how the students were grouped. Thus, the studies seem to align with traditional research designs focused on the medical and healthcare sector as well as to have a sound methodological foundation, although some methodological questions may be raised concerning the study by Cunningham and Roche [23].

## Conclusion

Finally, the reviewed articles draw on digital technologies such as web-based platforms, e-learning modules, calculators, simulation, PDAs, such as hand-held computers, among other devices. However, in the present review, we found no study focusing on, for instance, the use of virtual reality and head-mounted displays to create an immersive experience of MDC situations. Here, new technologies may bring new opportunities to enhance nursing students’ MDC skills globally.

## Supplementary Information

**Additional file 1.**

## Data Availability

The dataset from this study is available from the corresponding author upon reasonable request.

## Abbreviations

MDC: Medication dose calculation
ICT: Information and communications technology
PDA: Personal digital assistants
CL: Computerized learning
IL: Integrative learning
DIF: Demographic Information Form
WBI: Web-based instruction
DDC: Drug Dose Calculation
MSKA: The Medication Safety Knowledge Assessment
MSCEC: Medication Safety Critical Element Checklist was used to assess competency
