# Supplementary material for: Medication dosage calculation among nursing students: does digital technology make a difference? A literature review
Source: BMC Nurs. 2022 May 23;21:123. doi: 10.1186/s12912-022-00904-3 (PMC9125812; doi:10.1186/s12912-022-00904-3)
Supplement: Supplementary file 1 — Additional file 1. [file 12912_2022_904_MOESM1_ESM.docx]

**Supplementary material**

| Completion search Scopus 25 august 2021 | | | |
| --- | --- | --- | --- |
| No | Description |  | Items found (approx.) |
| 1 | Nursing student | TITLE-ABS-KEY("nurs* pupil" OR "nurs* diploma Program*" OR "nurs* educat*" OR "nurs* Program*" OR "nurs* student" OR "nurs* train*" OR "nurs* educat* research" OR "pupil nurs*" OR "student nurs*") | 122 645 |
| 2 | Drug dosage calculation | TITLE-ABS-KEY("administ* of medic*" OR "calcula* skill*" OR "dos* calcula* educat*" OR "dos* calcula* methods" OR "dos* calcula*" OR "dos* of drug*" OR "drug administ* educat*" OR "drug administ* nurs*" OR "drug administ*" OR "drug calcula* test*" OR "drug calcula*" OR "drug dos* calcula*" OR "drug prepar*" OR "mathem* skill*" OR "medic* calcula*" OR "medic* compet*" OR "medic* document*" OR "medic* dos* calcula*" OR "medic* error*" OR "medic* safe*" OR "numer* skill*") | 1 476 249 |
| 3 | ICT | TITLE-ABS-KEY ("personal digital assistant" OR mobile OR handheld OR calculator OR calculation OR "virtual reality" OR "educat* techn*" OR "nursing inform*" OR "comput* assist* instruct*" OR "comput* assist* learn*" OR multimedia OR simulat* OR e-learn* OR online OR "interact* learn*" OR digital OR "virtual learn*" OR "comp* based learn*" OR "comput* learn*" OR "game-based" OR "digit* game" OR "educat* game" OR "educat* strateg*" OR "electron* learn*" OR "information* technol*" OR "learn* labora*" OR "medici* game" OR "nurs* intervent*" OR "online medic*" OR "open learn*" OR "role-play" OR "self directed learn*" OR smartphone OR "web based" OR digital* OR computer* OR game OR "distance educat*" OR ICT OR "information* and commun* technolog*" OR interactive OR "non-tradit* educat*" OR "technol* Enhanced") | 12 515 528 |
| 4 |  | 1 AND 2 AND 3 | 564 |
| 5 |  | 4 AND Filters activated:  Publication type: Article  Language: English  Alla nyckelartiklar kommer med utom den som inte är indexerad samt en som är indexerad som en review. | 442 |

| Completion search Web of Science 25 august 2021 | | | |
| --- | --- | --- | --- |
| No | Description |  | Items found (approx.) |
| 1 | Nursing student | TS=( "nurs* pupil" OR "Nurs* diploma Program*" OR "nurs* educat*" OR "Nurs* Program*" OR "nurs* student" OR "nurs* train*" OR "nurs* educat* research" OR "pupil nurs*" OR "student nurs*" ) | 21 343 |
| 2 | Drug dosage calculation | TS=( "administ* of medic*" OR "calcula* skill*" OR "dos* calcula* educat*" OR "dos* calcula* methods" OR "dos* calcula*" OR "dos* of drug*" OR "drug administ* educat*" OR "drug administ* nurs*" OR "drug administ*" OR "drug calcula* test*" OR "drug calcula*" OR "drug dos* calcula*" OR "drug prepar*" OR "mathem* skill*" OR "medic* calcula*" OR "medic* compet*" OR "medic* document*" OR "medic* dos* calcula*" OR "medic* error*" OR "medic* safe*" OR "numer* skill*" OR "calcula* competen*" OR "math* competen*" OR "numer* competen*" ) | 90 511 |
| 3 | ICT | TS=("personal digital assistant" OR mobile OR handheld OR calculator OR calculation OR "virtual reality" OR "educat* techn*" OR "nursing inform*" OR "comput* assist* instruct*" OR "comput* assist* learn*" OR multimedia OR simulat* OR e-learn* OR online OR "interact* learn*" OR digital OR "virtual learn*" OR "comp* based learn*" OR "comput* learn*" OR "game-based" OR "digit* game" OR "educat* game" OR "educat* strateg*" OR "electron* learn*" OR "information* technol*" OR "learn* labora*" OR "medici* game" OR "nurs* intervent*" OR "online medic*" OR "open learn*" OR "role-play" OR "self directed learn*" OR smartphone OR "web based" OR digital* OR computer* OR game OR "distance educat*" OR ICT OR "information* and commun* technolog*" OR interactive OR "non-tradit* educat*" OR "technol* Enhanced") | 7 054 016 |
| 4 |  | 1 AND 2 AND 3 | 159 |
| 5 |  | 4 AND Filters activated:  Publication type: Article  Language: English  De åtta nyckelartiklar som är indexerade i databasen återvinns med sökstrategin. | 125 |

| Completion search Chinal 25 august 2021 | | | |
| --- | --- | --- | --- |
| No | Description |  | Items found (approx.) |
| 1 | Nursing student | TI( "nurs* pupil" OR "Nurs* diploma Program*" OR "nurs* educat*" OR "Nurs* Program*" OR "nurs* student" OR "nurs* train*" OR "nurs* educat* research" OR "pupil nurs*" OR "student nurs*" )  OR  AB( "nurs* pupil" OR "Nurs* diploma Program*" OR "nurs* educat*" OR "Nurs* Program*" OR "nurs* student" OR "nurs* train*" OR "nurs* educat* research" OR "pupil nurs*" OR "student nurs*" )  OR  MH("Students, Nursing" OR "Students, Nurse Midwifery" OR "Students, Nursing, Associate" OR "Students, Nursing, Baccaluareate" OR "Students, Nursing, Diploma Programs" OR "Students, Nursing, Graduate" OR "Students, Nursing, Masters" OR "Students, Nursing, Doctoral" OR "Students, Nursing, Male" OR "Students, Nursing, Practical" OR "Students, Pre-Nursing" OR Education, Nursing, Associate OR "Education, Nursing, Diploma Programs" OR "Education, Nursing, Practical" OR "Education, Nursing, Masters" OR "Education, Nursing, Doctoral" OR "Education, Nursing, Graduate" OR "Education, Nursing, Continuing" OR "Education, Nursing, Baccalaureate" OR "Education, Nursing" OR "Education, Nursing, Research-Based") | 111 174 |
| 2 | Drug dosage calculation | TI( "administ* of medic*" OR "calcula* skill*" OR "dos* calcula* educat*" OR "dos* calcula* methods" OR "dos* calcula*" OR "dos* of drug*" OR "drug administ* educat*" OR "drug administ* nurs*" OR "drug administ*" OR "drug calcula* test*" OR "drug calcula*" OR "drug dos* calcula*" OR "drug prepar*" OR "mathem* skill*" OR "medic* calcula*" OR "medic* compet*" OR "medic* document*" OR "medic* dos* calcula*" OR "medic* error*" OR "medic* safe*" OR "numer* skill*" OR "calcula* competen*" OR "math* competen*" OR "numer* competen*" )  OR  AB( "administ* of medic*" OR "calcula* skill*" OR "dos* calcula* educat*" OR "dos* calcula* methods" OR "dos* calcula*" OR "dos* of drug*" OR "drug administ* educat*" OR "drug administ* nurs*" OR "drug administ*" OR "drug calcula* test*" OR "drug calcula*" OR "drug dos* calcula*" OR "drug prepar*" OR "mathem* skill*" OR "medic* calcula*" OR "medic* compet*" OR "medic* document*" OR "medic* dos* calcula*" OR "medic* error*" OR "medic* safe*" OR "numer* skill*" OR "calcula* competen*" OR "math* competen*" OR "numer* competen*" )  OR  MH("Dosage Calculation" OR "dosage forms" OR "Drug administration" OR "drug compounding" OR "Medication errors" OR "Patient Safety" OR "Adverse Drug Event" OR "Medication Management") | 131 553 |
| 3 | ICT | TI("personal digital assistant" OR mobile OR handheld OR calculator OR calculation OR "virtual reality" OR "educat* techn*" OR "nursing inform*" OR "comput* assist* instruct*" OR "comput* assist* learn*" OR multimedia OR simulat* OR e-learn* OR online OR "interact* learn*" OR digital OR "virtual learn*" OR "comp* based learn*" OR "comput* learn*" OR "game-based" OR "digit* game" OR "educat* game" OR "educat* strateg*" OR "electron* learn*" OR "information* technol*" OR "learn* labora*" OR "medici* game" OR "nurs* intervent*" OR "online medic*" OR "open learn*" OR "role-play" OR "self directed learn*" OR smartphone OR "web based" OR digital* OR computer* OR game OR "distance educat*" OR ICT OR "information* and commun* technolog*" OR interactive OR "non-tradit* educat*" OR "technol* Enhanced")  OR  AB("personal digital assistant" OR mobile OR handheld OR calculator OR calculation OR "virtual reality" OR "educat* techn*" OR "nursing inform*" OR "comput* assist* instruct*" OR "comput* assist* learn*" OR multimedia OR simulat* OR e-learn* OR online OR "interact* learn*" OR digital OR "virtual learn*" OR "comp* based learn*" OR "comput* learn*" OR "game-based" OR "digit* game" OR "educat* game" OR "educat* strateg*" OR "electron* learn*" OR "information* technol*" OR "learn* labora*" OR "medici* game" OR "nurs* intervent*" OR "online medic*" OR "open learn*" OR "role-play" OR "self directed learn*" OR smartphone OR "web based" OR digital* OR computer* OR game OR "distance educat*" OR ICT OR "information* and commun* technolog*" OR interactive OR "non-tradit* educat*" OR "technol* Enhanced")  OR  MH("Computers, Hand-held" OR "Virtual reality" OR "Education, Non-Traditional" OR "Educational technology" OR "Nursing informatics" OR "Computer Assisted Instruction" OR Multimedia OR Simulations OR "computer simulation" OR "Online Education" OR "Digital Technology" OR Games OR "Information technology" OR "Learning Laboratories" OR "Nursing Interventions" OR "Role Playing" OR "Self Directed Learning" OR Smartphone OR "mobile applications" OR "cellular phone" OR "Computer Simulation" OR "Computerized Educational Testing") | 401 983 |
| 4 |  | 1 AND 2 AND 3 | 852 |
| 5 |  | 4 AND Filters activated:  Publication type: Academic journals  Language: English  Peer reviewed finns inte längre som en begränsning i Cinahl.  De tretton nyckelartiklar som är indexerade i databasen återvinns med sökstrategin. | 687 |

| Completion search Academic Search Premiere 25 august 2021 | | | |
| --- | --- | --- | --- |
| No | Description |  | Items found (approx.) |
| 1 | Nursing student | TI( "nurs* pupil" OR "Nurs* diploma Program*" OR "nurs* educat*" OR "Nurs* Program*" OR "nurs* student" OR "nurs* train*" OR "nurs* educat* research" OR "pupil nurs*" OR "student nurs*" )  OR  AB( "nurs* pupil" OR "Nurs* diploma Program*" OR "nurs* educat*" OR "Nurs* Program*" OR "nurs* student" OR "nurs* train*" OR "nurs* educat* research" OR "pupil nurs*" OR "student nurs*" )  OR  DE("NURSING students" OR "NURSING education" OR "NURSING schools" OR "ASSOCIATE degree nursing education" OR "BACCALAUREATE nursing education" OR "CONTINUING education of nurses" OR "GRADUATE nursing education" OR "PUBLIC health nursing education" OR "NURSING school graduates" OR "NURSING preceptorship") | 30 949 |
| 2 | Drug dosage calculation | TI( "administ* of medic*" OR "calcula* skill*" OR "dos* calcula* educat*" OR "dos* calcula* methods" OR "dos* calcula*" OR "dos* of drug*" OR "drug administ* educat*" OR "drug administ* nurs*" OR "drug administ*" OR "drug calcula* test*" OR "drug calcula*" OR "drug dos* calcula*" OR "drug prepar*" OR "mathem* skill*" OR "medic* calcula*" OR "medic* compet*" OR "medic* document*" OR "medic* dos* calcula*" OR "medic* error*" OR "medic* safe*" OR "numer* skill*" OR "calcula* competen*" OR "math* competen*" OR "numer* competen*" )  OR  AB( "administ* of medic*" OR "calcula* skill*" OR "dos* calcula* educat*" OR "dos* calcula* methods" OR "dos* calcula*" OR "dos* of drug*" OR "drug administ* educat*" OR "drug administ* nurs*" OR "drug administ*" OR "drug calcula* test*" OR "drug calcula*" OR "drug dos* calcula*" OR "drug prepar*" OR "mathem* skill*" OR "medic* calcula*" OR "medic* compet*" OR "medic* document*" OR "medic* dos* calcula*" OR "medic* error*" OR "medic* safe*" OR "numer* skill*" OR "calcula* competen*" OR "math* competen*" OR "numer* competen*")  OR  DE("Drug administration" OR "drug dosage" OR "Dosage forms of drugs" OR "Medication errors" OR "Patient Safety" OR "Medication therapy management" OR "nursing errors") | 170 313 |
| 3 | ICT | TI("personal digital assistant" OR mobile OR handheld OR calculator OR calculation OR "virtual reality" OR "educat* techn*" OR "nursing inform*" OR "comput* assist* instruct*" OR "comput* assist* learn*" OR multimedia OR simulat* OR e-learn* OR online OR "interact* learn*" OR digital OR "virtual learn*" OR "comp* based learn*" OR "comput* learn*" OR "game-based" OR "digit* game" OR "educat* game" OR "educat* strateg*" OR "electron* learn*" OR "information* technol*" OR "learn* labora*" OR "medici* game" OR "nurs* intervent*" OR "online medic*" OR "open learn*" OR "role-play" OR "self directed learn*" OR smartphone OR "web based" OR digital* OR computer* OR game OR "distance educat*" OR ICT OR "information* and commun* technolog*" OR interactive OR "non-tradit* educat*" OR "technol* Enhanced")  OR  AB("personal digital assistant" OR mobile OR handheld OR calculator OR calculation OR "virtual reality" OR "educat* techn*" OR "nursing inform*" OR "comput* assist* instruct*" OR "comput* assist* learn*" OR multimedia OR simulat* OR e-learn* OR online OR "interact* learn*" OR digital OR "virtual learn*" OR "comp* based learn*" OR "comput* learn*" OR "game-based" OR "digit* game" OR "educat* game" OR "educat* strateg*" OR "electron* learn*" OR "information* technol*" OR "learn* labora*" OR "medici* game" OR "nurs* intervent*" OR "online medic*" OR "open learn*" OR "role-play" OR "self directed learn*" OR smartphone OR "web based" OR digital* OR computer* OR game OR "distance educat*" OR ICT OR "information* and commun* technolog*" OR interactive OR "non-tradit* educat*" OR "technol* Enhanced")  OR  DE("Pocket computers" OR "Virtual reality" OR "nonformal education" OR "Educational technology" OR "Nursing informatics" OR "Computer Assisted Instruction" OR "Multimedia systems in education" OR "Simulation methods in education" OR "Simulation methods in higher education" OR "Simulation games in education" OR "Online Education" OR "Digital Technology" OR "educational Games" OR "nonformal Education" OR "Information technology" OR "Learning Laboratories" OR "Nursing Interventions" OR "Role Playing" OR Smartphones OR "mobile apps" OR "Computer Simulation" OR "Computer Assisted Instruction" OR "distance education" OR "drug administration software" OR "drug dosage software") | 3 416 439 |
| 4 |  | 1 AND 2 AND 3 | 224 |
| 5 |  | 4 AND Filters activated:  Publication type: Article, Academic journal, Scholarly (Peer Reviewed) Journals  Language: English  De två nyckelartiklar som är indexerade i databasen återvinns med sökstrategin. | 194 |
|  |  |  |  |

| Completion search ERIC 25 august 2021 | | | |
| --- | --- | --- | --- |
| No | Description |  | Items found (approx.) |
| 1 | Nursing student | TI( "nurs* pupil" OR "Nurs* diploma Program*" OR "nurs* educat*" OR "Nurs* Program*" OR "nurs* student" OR "nurs* train*" OR "nurs* educat* research" OR "pupil nurs*" OR "student nurs*" )  OR  AB( "nurs* pupil" OR "Nurs* diploma Program*" OR "nurs* educat*" OR "Nurs* Program*" OR "nurs* student" OR "nurs* train*" OR "nurs* educat* research" OR "pupil nurs*" OR "student nurs*" )  OR  DE("NURSING students" OR "NURSING education") | 6 463 |
| 2 | Drug dosage calculation | TI( "administ* of medic*" OR "calcula* skill*" OR "dos* calcula* educat*" OR "dos* calcula* methods" OR "dos* calcula*" OR "dos* of drug*" OR "drug administ* educat*" OR "drug administ* nurs*" OR "drug administ*" OR "drug calcula* test*" OR "drug calcula*" OR "drug dos* calcula*" OR "drug prepar*" OR "mathem* skill*" OR "medic* calcula*" OR "medic* compet*" OR "medic* document*" OR "medic* dos* calcula*" OR "medic* error*" OR "medic* safe*" OR "numer* skill*" OR "calcula* competen*" OR "math* competen*" OR "numer* competen*" )  OR  AB( "administ* of medic*" OR "calcula* skill*" OR "dos* calcula* educat*" OR "dos* calcula* methods" OR "dos* calcula*" OR "dos* of drug*" OR "drug administ* educat*" OR "drug administ* nurs*" OR "drug administ*" OR "drug calcula* test*" OR "drug calcula*" OR "drug dos* calcula*" OR "drug prepar*" OR "mathem* skill*" OR "medic* calcula*" OR "medic* compet*" OR "medic* document*" OR "medic* dos* calcula*" OR "medic* error*" OR "medic* safe*" OR "numer* skill*" OR "calcula* competen*" OR "math* competen*" OR "numer* competen*" ) | 3 810 |
| 3 | ICT | TI("personal digital assistant" OR mobile OR handheld OR calculator OR calculation OR "virtual reality" OR "educat* techn*" OR "nursing inform*" OR "comput* assist* instruct*" OR "comput* assist* learn*" OR multimedia OR simulat* OR e-learn* OR online OR "interact* learn*" OR digital OR "virtual learn*" OR "comp* based learn*" OR "comput* learn*" OR "game-based" OR "digit* game" OR "educat* game" OR "educat* strateg*" OR "electron* learn*" OR "information* technol*" OR "learn* labora*" OR "medici* game" OR "nurs* intervent*" OR "online medic*" OR "open learn*" OR "role-play" OR "self directed learn*" OR smartphone OR "web based" OR digital* OR computer* OR game OR "distance educat*" OR ICT OR "information* and commun* technolog*" OR interactive OR "non-tradit* educat*" OR "technol* Enhanced")  OR  AB("personal digital assistant" OR mobile OR handheld OR calculator OR calculation OR "virtual reality" OR "educat* techn*" OR "nursing inform*" OR "comput* assist* instruct*" OR "comput* assist* learn*" OR multimedia OR simulat* OR e-learn* OR online OR "interact* learn*" OR digital OR "virtual learn*" OR "comp* based learn*" OR "comput* learn*" OR "game-based" OR "digit* game" OR "educat* game" OR "educat* strateg*" OR "electron* learn*" OR "information* technol*" OR "learn* labora*" OR "medici* game" OR "nurs* intervent*" OR "online medic*" OR "open learn*" OR "role-play" OR "self directed learn*" OR smartphone OR "web based" OR digital* OR computer* OR game OR "distance educat*" OR ICT OR "information* and commun* technolog*" OR interactive OR "non-tradit* educat*" OR "technol* Enhanced")  OR  DE("Distance education" OR "Educational technology" OR "Computer Assisted Instruction" OR "Multimedia material" OR Multimedia instruction" OR "Computer simulation" OR " Nontraditional Education" OR "Computer games" OR "Information technology" OR "Learning Laboratories" OR "Role Playing" OR "Independent study" OR "Handheld devices" OR "Computer Assisted Instruction" OR "Computer Uses in Education" OR "open source technology" OR Computers OR "online courses" OR "web based instruction" OR "electronic learning") | 248 880 |
| 4 |  | 1 AND 2 AND 3 | 27 |
| 5 |  | 4 AND Filters activated:  Publication type: Academic journal, Peer Reviewed  Language: English  Inga ämnesord för läkemedelsberäkning.  De två nyckelartiklar som är indexerade i databasen återvinns med sökstrategin. | 10 |
|  |  |  |  |

| Completion search PubMed 25 august 2021 | | | |
| --- | --- | --- | --- |
| No | Description |  | Items found (approx.) |
| 1 | Nursing student | nursing pupil*[tiab] OR nurse student*[tiab] OR nursing educat*[tiab] OR nurse educat*[tiab] OR Nursing Program*[tiab] OR nurse program*[tiab] OR nursing student*[tiab] OR nurse student*[tiab] OR nursing train*[tiab] OR nurse train*[tiab] OR pupil nurs*[tiab] OR student nurs*[tiab]  OR  "Students, Nursing"[mesh] OR "Students, Public Health"[mesh] OR "Students, Health Occupations"[mesh] OR "Students, Premedical"[mesh] OR "Education, Nursing"[mesh] OR "Education, premedical"[mesh] OR "Education, Nursing, Graduate"[mesh] OR "Education, Nursing, Continuing"[mesh] OR "Education, Nursing, Baccalaureate"[mesh] OR "Competency-Based Education "[mesh] OR "Education, Nursing, Associate"[mesh] OR "Education, Nursing, Diploma Programs"[mesh] OR "nursing education research"[mesh] | 161 031 |
| 2 | Drug dosage calculation | administration of medic*[tiab] OR calculation skill*[tiab] OR dosage calculation*[tiab] OR dosage of drug*[tiab] OR drug administrat*[tiab] OR drug calcula*[tiab] OR drug dos*[tiab] OR drug prepar*[tiab] OR mathematical skill*[tiab] OR medication calcula*[tiab] OR medical compet*[tiab] OR medical document*[tiab] OR medication error*[tiab] OR medical safe*[tiab] OR numerical skill*[tiab] OR calculation competen*[tiab] OR mathematical competen*[tiab] OR numeracy competen*[tiab]  OR  "Drug Dosage Calculations"[mesh] OR "Drug compounding"[mesh] OR "Medication errors"[mesh] OR "Patient Safety"[mesh] OR "Drug-Related Side Effects and Adverse Reactions"[mesh] OR mathematics[mesh] | 456 859 |
| 3 | ICT | personal digital assistant*[tiab] OR mobile[tiab] OR handheld[tiab] OR calculator[tiab] OR calculation[tiab] OR virtual reality[tiab] OR educational techn*[tiab] OR nursing inform*[tiab] OR computer assisted[tiab] OR multimedia[tiab] OR simulat*[tiab] OR e-learn*[tiab] OR online[tiab] OR interactive learn*[tiab] OR digital[tiab] OR virtual learn*[tiab] OR computer based learn*[tiab] OR computer learn*[tiab] OR game-based[tiab] OR digital game*[tiab] OR educational game*[tiab] OR educational strateg*[tiab] OR electronic learn*[tiab] OR information technol*[tiab] OR learning labora*[tiab] OR medical game*[tiab] OR nursing intervent*[tiab] OR nurse intervent*[tiab] OR online medic*[tiab] OR open learn*[tiab] OR role-play[tiab] OR self directed learn*[tiab] OR smartphone*[tiab] OR web based[tiab] OR digital*[tiab] OR computer*[tiab] OR game*[tiab] OR distance educat*[tiab] OR ICT[tiab] OR information and communication technolog*[tiab] OR interactive[tiab] OR non-traditional educat* [tiab] OR technology Enhanced[tiab]  OR  "Computers, Handheld"[mesh] OR "Virtual reality"[mesh] OR "Educational technology"[mesh] OR "Nursing informatics"[mesh] OR "Multimedia"[mesh] OR "computer Simulation"[mesh] "simulation training"[mesh] OR "high fidelity simulation training"[mesh] OR "Digital Technology"[mesh] OR "Information technology"[mesh] OR "Role Playing"[mesh] OR "Self-Directed Learning as Topic"[mesh] OR "Smartphone"[mesh] OR "Computer-Assisted Instruction"[mesh] | 1 529 412 |
| 4 |  | 1 AND 2 AND 3 | 688 |
| 5 |  | 4 AND Filters activated:  Publication type: Journal article  Language: English  Tretton av de fjorton nyckelartiklar som är indexerade i databasen återvinns med sökstrategin. | 653 |
